# Genetic Pleiotropy Explains Associations between Musical Auditory Discrimination and Intelligence

**DOI:** 10.1371/journal.pone.0113874

**Published:** 2014-11-24

**Authors:** Miriam A. Mosing, Nancy L. Pedersen, Guy Madison, Fredrik Ullén

**Affiliations:** 1 Department of Neuroscience, Karolinska Institutet, Stockholm, Sweden; 2 Department of Medical Epidemiology and Biostatistics, Karolinska Institutet, Stockholm, Sweden; 3 Department of Psychology, Umeå University, Umeå, Sweden; UNLV, United States of America

## Abstract

Musical aptitude is commonly measured using tasks that involve discrimination of different types of musical auditory stimuli. Performance on such different discrimination tasks correlates positively with each other and with intelligence. However, no study to date has explored these associations using a genetically informative sample to estimate underlying genetic and environmental influences. In the present study, a large sample of Swedish twins (N = 10,500) was used to investigate the genetic architecture of the associations between intelligence and performance on three musical auditory discrimination tasks (rhythm, melody and pitch). Phenotypic correlations between the tasks ranged between 0.23 and 0.42 (Pearson *r* values). Genetic modelling showed that the covariation between the variables could be explained by shared genetic influences. Neither shared, nor non-shared environment had a significant effect on the associations. Good fit was obtained with a two-factor model where one underlying shared genetic factor explained all the covariation between the musical discrimination tasks and IQ, and a second genetic factor explained variance exclusively shared among the discrimination tasks. The results suggest that positive correlations among musical aptitudes result from both genes with broad effects on cognition, and genes with potentially more specific influences on auditory functions.

## Introduction

Musical aptitude is commonly measured with tests tapping into the ability to discriminate musical auditory stimuli, e.g. melodies, rhythms, and pitches [1]. Following the pioneering work by Seashore [2], [3] many such tests have been developed for various purposes [4]–[10]. Moderate positive correlations have typically been reported between individual test scales measuring discrimination skills of different types of musical stimuli [5]–[7] suggesting the existence of an underlying more general musicality factor [11]. In line with this, discrimination of musical stimuli correlates with self-report measures of general musical sophistication and engagement [12]. Also, it consistently has been shown that musical aptitudes correlate positively with intelligence [13]–[15]. This correlation between intelligence and sensory discrimination is not unique for musical stimuli but extends to a variety of stimulus attributes in different sensory modalities. Examples include timing [16], brightness [17], hue [18], and colour [19] of visual stimuli; tactile/kinesthetic stimuli providing information about the roughness, shape, and weight of objects, as well as the position and trajectory of body parts [18], [20], [21]; and timing [16], [17], pitch [17], [18], and intensity [17] of auditory stimuli. Sensory discrimination and acuity decrement during aging and, interestingly, these changes in perceptual processing may have a causal influence on age-related decrements in intelligence and performance on cognitive tasks, such as short-term memory [22], [23].

Interest in the relation between cognition and sensory discrimination in general cognitive ability research dates back to Galton (1883) [24], who was the first to suggest such an association. Later, Spearman [18] – on the basis of observed correlations between intelligence and pitch, weight and hue discrimination – hypothesized a potentially perfect association between ‘general sensory discrimination’ and general intelligence. More recently, Troche and Rammsayer [17] found some support for the existence of a general discrimination factor using structural equation modelling, reporting good fit for a model with a single, latent general discrimination ability factor mediating the associations between various sensory discrimination tasks and intelligence. However, Spearman's idea of a perfect association between general discrimination ability and intelligence appears untenable, given the consistently modest correlations between discrimination and intelligence found in recent studies.

To our knowledge, the present study is the first to use a genetically informative sample - a large cohort of Swedish twins - to explore associations between sensory discrimination and intelligence (IQ). Specific aims were: (1) to estimate genetic and environmental influences underlying the associations between three different musical auditory discrimination skills - i.e. melody, rhythm, and pitch perception - and IQ to establish whether the phenotypic correlations between the variables depend on genetic pleiotropy (i.e. genetic effects common to discrimination ability and IQ); (2) to investigate whether the covariance between all four variables could entirely be explained by one ‘general cognitive ability factor’, as suggested by Spearman, or whether a two-factor model with an additional musical discrimination factor explaining some of the covariance between the musicality tests provides a better overall fit.

## Methods

### Participants

Data collection was based on a web-survey sent out to the STAGE cohort, part of the Swedish Twin Registry [25], between 2012 and 2013. For further details on the web survey see [7]. Zygosity was determined based on questions about intra-pair resemblance. In the Swedish Twin Registry, this method has been shown to be more than 98% accurate when zygosity status was confirmed using genotyping [26]. The study was conducted according to the principles expressed in the Declaration of Helsinki, was approved by the Regional Ethics Review Board in Stockholm (Dnr 2011/570-31/5, 2012/1107/32), and informed written consent was obtained from all participants. Participants were compensated with one movie ticket for their participation. The final sample consisted of 10,537 participants aged 27–54 (mean 40.7, SD 7.7) with a score for at least one of the traits studied here. The sample was comprised of 2,568 complete twin pairs (1,210 monozygotic (MZ) and 1,358 dizygotic (DZ) same- and opposite-sex twin pairs) and 5,401 single twins without a participating co-twin. Single twins were included as they contribute to the estimation of means, variances, and covariance effects.

#### Musical ability

Musical ability was measured using the Swedish Musical Discrimination Test (SMDT) [7]. The SMDT is similar in construction to the Seashore test and was designed (i) for online administration, (ii) to minimize test time, and (iii) to have a suitable difficulty level for modern populations in Western countries. Earlier analyses have shown that the SMDT has good psychometric properties. Cronbach's alpha values and Spearman–Brown split-half reliabilities for the subtests ranged between 0.79 and 0.89. Criterion validity has been demonstrated in several ways: individuals which have played a musical instrument score higher than individuals that have not, musically educated individuals score higher than individuals who have not taken music lessons, and SMDT scores correlate with hours of musical training [7], [27]. The three sub-tests are outlined here. For a detailed description and psychometric validation, see [7]. *Pitch* consisted of 27 trials. In each trial, two successive sine tones were presented. The tones had a duration of 590 ms and were separated by 1 s of silence. The frequency of one of the tones was always 500 Hz, whereas the frequency of the other tone varied in the range 501–517 Hz, i.e. the difference between the tones was 3.5–57.9 cents. Participants were instructed to indicate whether the first tone was higher or lower in pitch than the second tone. Responses were given either by pressing the ‘H’ or ‘L’ key on keyboard, or by clicking on corresponding icons with the mouse pointer. Item difficulty (i.e. the difference in pitch between the two tones) increased progressively. *Melody* included 18 trials. Stimuli consisted of sequences of piano tones with pitches ranging from C4 to A#5 (American Standard Pitch; 262 to 932 Hz) and a constant time interval between tones of 650 ms. In each trial, two stimuli were presented, separated by 1.3 s of silence. The tone sequences of each stimulus were generated randomly with the constraints that each pitch occurred only once, not all pitches belonged to the same tonal (i.e., major, ascending minor, descending minor, or harmonic minor) scale, and all intervals between tones were smaller than one octave. The stimuli of a trial were identical, except for that the pitch of one randomly selected tone was altered in the second stimulus. This alternation was made so that the melodic contour (i.e. the pitch interval directions) did not change. The task of the participant was to indicate which tone had been changed. For this purpose, the sequences were graphically represented as a line of dots which changed colour when the corresponding tone was played. Responses were given by pressing a key corresponding to the differing note, or by clicking on the corresponding dot with the mouse pointer. Item difficulty was increased progressively by increasing the number of tones in a sequence from four to nine. In *Rhythm* (18 trials), stimuli consisted of two rhythmic sequences of sine tones with a duration of 60 ms. Inter-onset intervals between tones in a stimulus were always either 150, 300, 450, or 600 ms, i.e. only integer multiples of 150 ms were used. The two sequences of a trial were separated by 1 s of silence. In 11 out of the 18 Rhythm item the two stimuli differed; in the remaining items they were identical. Stimulus pairs within the same item typically differed in that one note was moved in time in the second stimulus as compared to the first stimulus, and/or that a different starting point in the sequence was used in the second stimulus. The task of the participant was to indicate whether the stimuli were the same or different, by either pressing one of two keys on the keyboard or by clicking one of two icons with the mouse pointer. The number of sounds in a stimulus sequence increased from five to seven as the subtest progressed. For all three subtests – Pitch, Melody and Rhythm - scores were calculated by adding up the number of correct trials.

#### Intelligence

Psychometric intelligence (IQ) was measured with the WMT [28]. The WMT is a visual matrix test similar in construction to Raven's standard progressive matrices (SPM) and has been shown to possess relatively high reliability (Cronbach's alpha = 0.81) and to correlate highly with Raven's SPM (*r* = 0.92) [28]. The test consists of 24 multiple choice items. The total score is a sum score with correctly answered items scored as one and incorrect or missing items scored as zero. Participants were given 25 minutes to complete the test, as detailed in the manual [28].

### Analyses and genetic modelling

**Figure 1:**
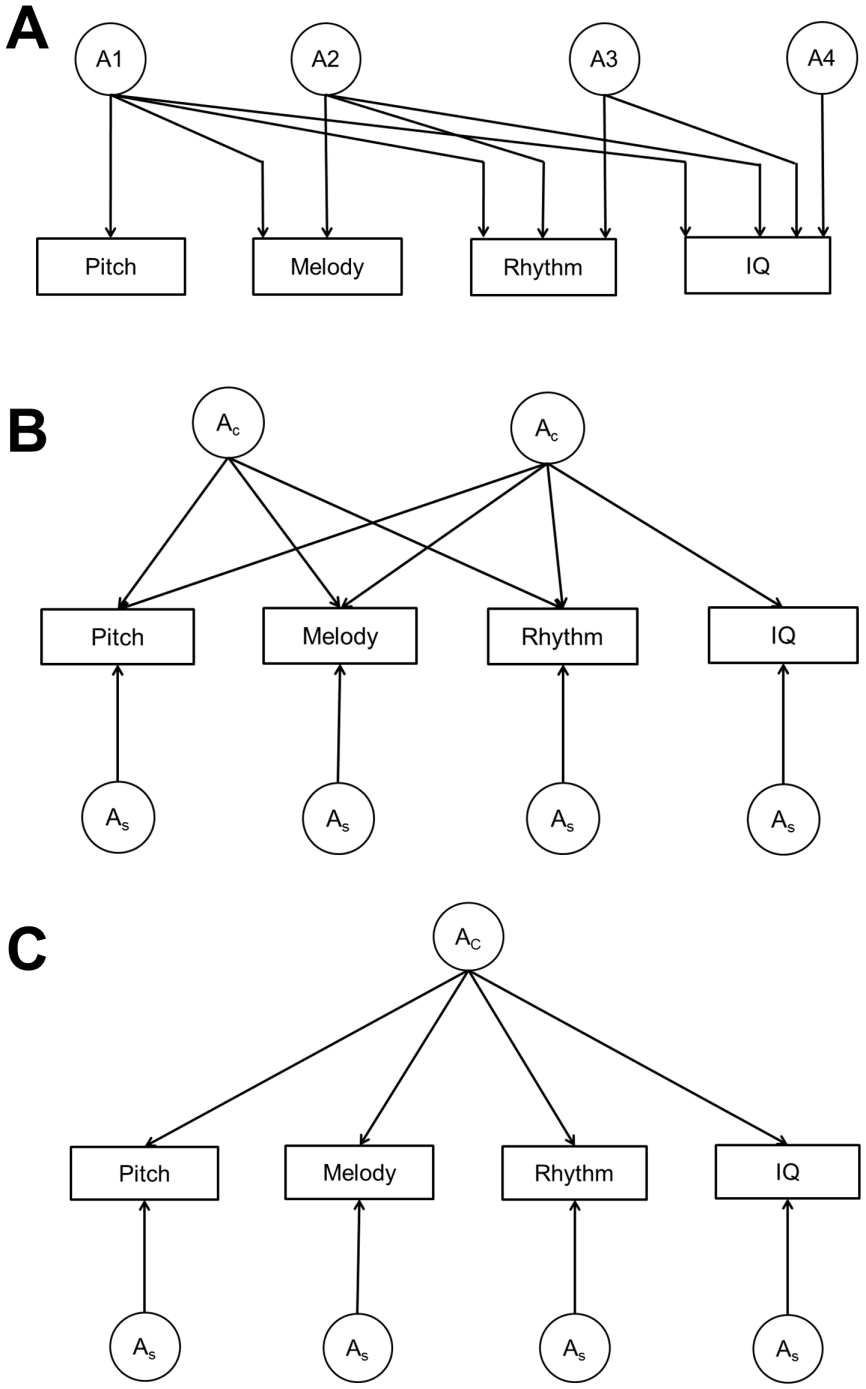
Different models applied to the data. (A) Cholesky decomposition. (B) Independent pathway model with two common genetic factors. (C) Independent pathway model with one common genetic factor. A = additive genetic influences. Subscripts *c* and *s* denote common and specific influences, respectively. Genetic factors of the Cholesky decomposition are labelled A1, A2, A3, and A4.

The three discrimination tasks as well as the IQ score were converted to z-scores. The genetic and environmental architecture of the covariation between the auditory discrimination tasks and IQ was analysed using the classical twin design. While MZ twins share all their genetic make-up, DZ twins only share half of their segregating genes on average. This information can be used to partition the variance within and between traits into that due to additive genetic (A), shared (C), and non-shared (E) environmental influences. Univariate sex-limitation analyses of the SMDT scores were fitted first and have been reported elsewhere [7]. As we were specifically interested in covariation between the traits, a multivariate ACE Cholesky decomposition (Figure 1A) was fitted first, using maximum-likelihood modeling in the structural equation modelling program Mx [29]. We have previously studied the nature of the association between music practice and music discrimination in the same cohort [27]. Since those analyses demonstrated that the associations were essentially driven by genetic pleiotropy, with no causal effect of practice on discrimination ability, we did not include music practice as a covariate in the present analyses. The variables were put in the model in the following order: Pitch, Melody, Rhythm, and IQ. Subsequently, the model fit of reduced models was compared to the full Cholesky decomposition to determine the model that fitted the data best. Finally, to test to what extent the associations between the traits were due to shared genetic etiology, two independent models were fitted to the data, with two and one common genetic A-factor, respectively (Figure 1B–C). The two factor model forces all genetic covariance to be shared between either the three discrimination tasks (common discrimination factor) or between the discrimination factor and IQ (cognitive ability factor) while at the same time allowing for specific genetic variance on each variable. This model would fit the data best if there was one genetic auditory discrimination factor that would be tapped by the three tasks with some additional genetic influences shared with IQ. The one factor solution forces all genetic covariance to be explained by one shared genetic factor with additional specific variance on each variable. This would be the best fitting model if the genetic aetiology underlying the relationship between auditory discrimination and IQ was entirely due to one set of shared genes (e.g. general cognitive ability).

## Results

### Preliminary analyses

Sex had a significant effect on IQ (t(8479) = 10.97, *p*<0.001, Cohen's *d* = 0.24), with men scoring slightly higher (M = 13.51, SE = 0.09) than females (M = 12.25, SE = 0.07), and on Pitch (t(6715) = 6.98, *p*<.001, Cohen's *d* = 0.17), with females scoring slightly lower (M = 17.80, SE = 0.07) than males (M = 18.65, SE = 0.10). Sex had no effect on the other two musical skills. Further, age showed a significant mean effect on IQ (*β* = −.93, t(8479) = −16.71, p<.001), with lower IQ with increased age, and on Pitch (*β* = −0.06, t(6715) = −5.06, *p*<.001) and Rhythm (*β* = −0.11, t(6878) = −9.21, *p*<.001), with lower pitch and rhythm discrimination skills with increased age. Sex and age were included as covariates in all further twin models. Descriptive statistics of the four variables are shown in Table 1.

**Table 1:** Descriptive statistics.

	Males (N = 2823*)			Females (N = 3840*)		
	N	M (SD)	Range	N	M (SD)	Range
Musical ability						
Rhythm	2920	15.4 (2.2)	4–18	3960	15.3 (2.2)	5–18
Melody	2875	6.8 (3.0)	0–18	3911	6.6 (2.8)	0–18
Pitch	2841	18.6 (5.1)	1–27	3876	17.8 (4.6)	1–27
IQ	3573	13.5 (5.4)	0–24	4908	12.3 (5.1)	0–24

*Note.* M = Mean; SD = standard deviation.

*participants who completed all four tests.

### Phenotypic associations

Table 2 shows the phenotypic (upper half) and twin correlations (lower half) corrected for age. The phenotypic correlations were all significant and moderate ranging between 0.23–0.42, with similar estimates for females and males. DZ twin correlations were more than half the MZ twin correlations suggesting an ACE model would fit the data best. Although twin correlations for Pitch and Melody suggested potential sex-limitation, with male DZ twins showing similar correlations to male MZ twins, previous univariate (general and common) sex-limitation analyses [7] only suggested sex-differences in the genetic etiology of pitch, but not in melody, rhythm or IQ. Therefore, for multivariate analyses (for ease of modeling) male and female pairs were analyzed together (MZ and DZ pairs) and variables were corrected for mean effects (sex and age).

**Table 2:** Phenotypic correlations (top) for females (below diagonal) and males (above diagonal) and twin correlations for each zygosity (bottom) for WMT, Rhythm, Pitch, and Melody corrected for sex and age.

	Phenotypic correlations (95% confidence intervals)			
	WMT	Rhythm	Melody	Pitch
WMT	-	0.29 (0.25; 0.32)	0.27 (0.23; 0.31)	0.29 (0.25; 0.32)
Rhythm	0.28 (0.25; 0.31)	-	0.42 (0.39; 0.45)	0.32 (0.29; 0.36)
Melody	0.23 (0.20; 0.26)	0.38 (0.35; 0.40)	-	0.41 (0.38; 0.44)
Pitch	0.23 (0.19; 0.26)	0.34 (0.31; 0.36)	0.37 (0.34; 0.40)	-

Zygosity	Twin correlations (95% confidence intervals)			
MZ	0.58 (0.54; 0.62)	0.51 (0.48; 0.56)	0.57 (0.52; 0.61)	0.48 (0.42; 0.53)
DZ	0.32 (0.27; 0.38)	0.28 (0.21; 0.35)	0.32 (0.25; 0.38)	0.29 (0.21; 0.35)
MZF	0.58 (0.53; 0.63)	0.52 (0.45; 0.58)	0.59 (0.53; 0.64)	0.45 (0.43; 0.52)
MZM	0.59 (0.52; 0.65)	0.50 (0.39; 0.58)	0.53 (0.43; 0.60)	0.51 (0.41; 0.59)
DZF	0.35 (0.25; 0.44)	0.27 (0.14; 0.39)	0.25 (0.13; 0.36)	0.33 (0.20; 0.44)
DZM	0.38 (0.25; 0.48)	0.30 (0.12; 0.45)	0.45 (0.30; 0.56)	0.49 (0.36; 0.60)
DZOS	0.27 (0.18; 0.35)	0.28 (0.17; 0.38)	0.29 (0.18; 0.39)	0.17 (0.06; 0.27)

Note: MZ = Monozygotic; DZ = Dizygotic; DZOS = DZ opposite-sex; F = Female; M = Male; WMT = Wiener Matrizen Test.

### Genetic analyses

**Figure 2:**
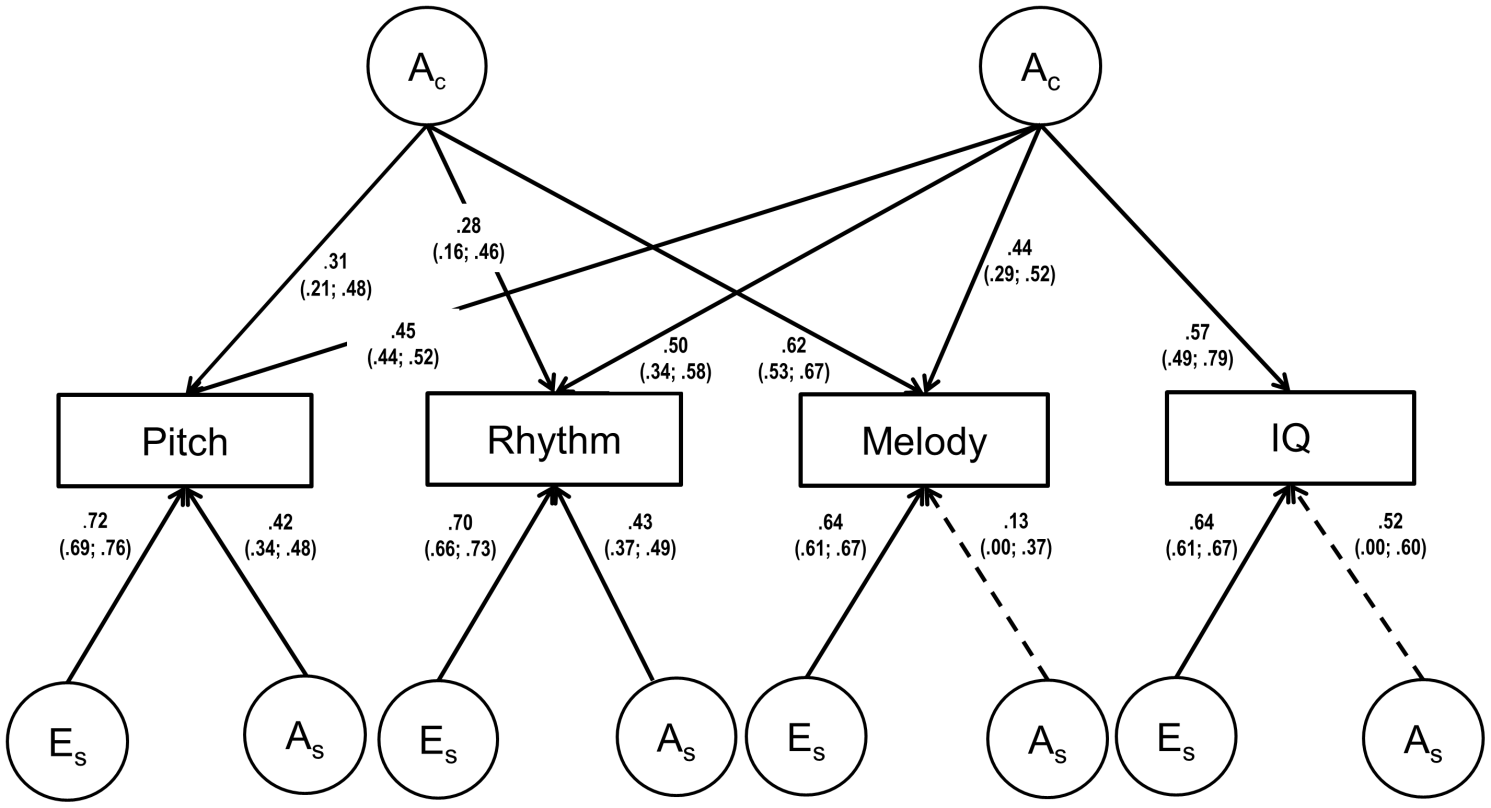
The reduced AE independent pathway model with two common genetic factors. Numbers represent path coefficients with confidence intervals within parentheses. Significant pathways are shown with solid lines; non-significant pathways are represented as dashed lines. A = additive genetic influences; E = non-shared environmental influences. Subscripts *c* and *s* denote common and specific influences, respectively.

Multivariate modelling results are shown in Table 3. All shared environmental (C) influences could be removed without a significant deterioration of model fit. Next, all E cross-paths could be removed, suggesting that all the covariance between the variables could be explained by shared genetic influences. Therefore, in the independent models all covariance was also explained by shared genetic influences (two or one common A-factors, respectively), allowing for additional specific A and E influences on each variable. Based on AIC and BIC, the two common factor solution (Figure 2) fitted the data almost as good as the reduced Cholesky, while the one common factor solution fitted the data significantly worse. Heritability estimates can be calculated by squaring and adding up all genetic pathways leading to one trait (Figure 2) – e.g. for Pitch: 0.31^2^+0.45^2^+0.42^2^ = 0.48 – showing that 48% (Pitch), 51% (Rhythm), 59% (Melody) and 60% (IQ) of the variance was due to genetic influences for these four traits. Note that the estimates here are slightly higher compared to those previously reported for the musical abilities in the same sample as they are based on the multivariate AE model where the non-significant variance originally explained by C has shifted to A [7]. As can be seen in Figure 2, the genetic factor shared with IQ explained approximately 42%, 49%, and 32% of the genetic variance for Pitch, Rhythm, and Melody, respectively, while about 20% (Pitch), 15% (Rhythm), and 64% were due to the genetic factor only shared among the discrimination abilities and, finally, the remainder was due to specific genetic influences. Further, shared genetic influences with the musical abilities explained about half of the genetic variance in IQ (32% of the total variance) with the remainder being due to a specific genetic factor (though non-significant).

**Table 3:** Multivariate model fitting results for the three music aptitude measures (Pitch, Rhythm, and Melody) and WMT (last) corrected for age and sex with the best fitting models highlighted in bold.

	AIC	BIC	-2LL	df	Δ -2LL	Δ - df	p-value
Cholesky decomposition – ACE	18427.74	−85128.15	74171.74	27872			
Cholesky decomposition – AE	18413.93	−85168.90	74177.93	27882	6.18	10	0.80
**Cholesky decomposition – AE (no E cross path)**	**18407.10**	**−85192.62**	**74183.10**	**27888**	**5.18**	**6**	**0.52**
**Independent model 2 common A factors – AE** *	**18409.10**	**−85188.24**	**74183.10**	**27887**	**-**	**-**	**-**
Independent model 1 common A factor – AE*	18447.31	−85179.29	74227.31	27890	44.21	3	<0.01

Note: A = additive genetic; C = common/shared environmental; E = non-shared environmental.

*E cross-paths are removed from the model.

Note that the independent model and the Cholesky decomposition are not nested and therefore cannot be directly compared.

## Discussion

### Genetic correlations between musical discrimination and intelligence

We studied the genetic architecture of associations between musical discrimination ability, measured with the SMDT, and intelligence. In line with earlier studies, our findings suggest moderate positive associations among the different musical auditory discrimination tasks [5]–[7], as well as between the musical tasks and intelligence [13]–[15]. These phenotypic associations appeared to be mainly due to genetic pleiotropy, with no significant contributions of non-genetic factors. The two-factor independent pathway model fitted the data as well as the reduced Cholesky decomposition, indicating that there was a single genetic factor explaining all the covariation between intelligence and discrimination, rather than separate genetic factors underlying associations between individual discrimination tasks and intelligence.

Univariate twin modelling of the musical tasks in the same dataset [7] has shown significant influences of genes – suggesting moderate heritabilities ranging between 12% and 59% – but no significant effect of shared environment (with the exception of pitch discrimination in males). Non-significant shared environmental effects on musical aptitude were also reported by Drayna and coworkers in a sample of adult female twins [30]. Interestingly, in adult participants, variation in family environment and other shared environmental factors thus appear to have little or no importance for individual differences in musical aptitudes, as well as for covariation of musical aptitudes with each other and with intelligence.

A significant part of the genetic variance was shared between the musical tasks and intelligence. This indicates that genetic influences on musical discrimination in part involve genes that influence not only musical but also non-musical cognitive tasks. Musical discrimination involves on-line manipulation of musical information in working memory. One possible explanation for the correlation between intelligence and musical discrimination is therefore that individual differences in attention, working memory and other executive functions related to intelligence influence both musical discrimination and performance in other cognitive tasks. In the present study, the highest load on working memory is presumably found in the Melody and Rhythm tests, which require discrimination between stimuli consisting of sequences of sounds. Similarly, earlier studies on discrimination and intelligence have included tasks such as Temporal Generalization and Rhythm Perception that also involve the processing of sequential structures in working memory [17], [31]. Intelligence is highly heritable [32] and shows substantial correlations with working memory and attention [33], [34]. Furthermore, there are strong genetic influences on executive functions as well as on their covariation with intelligence [35], [36].

Notably, however, associations between intelligence and sensory discrimination are also found for tasks with relatively low load on working memory. The Pitch subtest employed here involved stimuli consisting of single tones. Similarly, Troche and Rammsayer have earlier found associations between intelligence and discrimination of e.g. the brightness or the duration of simple, non-sequential stimuli [17]. Furthermore, intelligence is associated with inspection time, i.e. visual discrimination speed, and this association has been found to have a substantial genetic component in several studies [37]–[40]. These findings suggest that the association between intelligence and discrimination could also reflect basic neural properties that influence both the accuracy of early sensory processing and performance on cognitive tasks through bottom-up mechanisms. Notably, however, Troche and coworkers recently found good fit for a model where the association between simple discrimination tasks, that only involved the comparison of two non-sequential stimuli, and intelligence was mediated by working memory [41].

As summarized in the Introduction, many studies have documented phenotypic associations between intelligence and accuracy of discrimination of various types of sensory stimuli in different modalities. To our knowledge no previous studies have analysed the genetic architecture of associations between auditory discrimination and intelligence. From a neurobiological, ‘bottom up’ perspective it appears likely that the covariation between intelligence and musical aptitude involves global brain properties of broad importance for cognitive function, such as brain volume, white matter integrity and cortical thickness [42]. Many studies have found a moderate positive correlation between total brain volume and intelligence [42], [43], [44]. The relation appears to be entirely pleiotropic in nature [45]. Studies using measures of regional brain volume or cortical thickness have demonstrated anatomically widespread correlates of intelligence in the frontal, parietal and temporal lobes [42], [46], [47], and similarly musical aptitude has been shown to correlate positively with regional volume of the auditory cortex in the temporal lobe [48]. Conceivably, genes influencing global brain size could thus cause correlations between intelligence and musical perception, by simultaneously influencing the regional volume or organization of distributed sets of brain regions involved in these functions.

### Genetic effects on musical discrimination

While the two-factor model fit the data well, the one-factor solution did not fit, suggesting that there was genetic variance that was shared among the discrimination tasks but not with IQ. This ‘discrimination’ factor may represent more specific genetic influences on the auditory system. A recent genome-wide linkage and association study of musical aptitude in a Finnish population identified several single nucleotide polymorphisms close to genes known to be involved in auditory functions [49]. The strongest association was found at chromosomal locus 3q21.3, near the gene coding for GATA binding protein 2, which is involved in the developmental regulation of cochlear hair cells and the inferior colliculus. Musical aptitude has also been associated with genes for protocadherins, which are cell-adhesion proteins involved in neural development and neuroplastic processes [49], [50]. Oikkonen and coworkers found associations between musical aptitude and the protocadherin 7 gene, which is expressed in the cochlea, as well as the protocadherin 15 gene, which is essential for hair cell signal transduction [49]. Analyses of gene copy number variations have found low musical aptitude to co-segregate with a deletion at locus 5q31.1, which covers the protocadherin-α gene cluster [50].

Musical aptitude is correlated with linguistic abilities, including phonological awareness, pronunciation, and reading, even when controlling for intelligence [15], [51], [52], [53]. This suggests that genetic effects on musical aptitude may influence linguistic functions, over and above their effects on intelligence. In line with this, musical aptitude has been linked to locus 18q, which overlaps with the DYX6 locus associated with dyslexia [54], [55]. An interesting possibility is that specific genetic influences on musical aptitude could be implicated in clinical populations where musical and general cognitive abilities are dissociated, e.g. musical savants with high musical aptitude in spite of low intelligence, and amusic individuals with highly specific deficits in music perception [15].

Musical discrimination correlates positively with musical training [15]. We have recently studied the nature of this association, in the same participant cohort as here, using a combination of classical twin modelling and a monozygotic intrapair difference model [27]. Key findings of the study were that associations between training and musical discrimination were essentially caused by genetic pleiotropies, and that the association between the two measures disappeared entirely when all genetic and shared environmental factors were controlled for in the intrapair difference design. These results strongly speak against a causal effect of musical training on musical discrimination. Rather, they suggest that a common set of genes influences both musical discrimination and the tendency to engage in musical practicing. It appears likely, therefore, that the pleiotropic effects on musical discrimination and intelligence described in the present involve genes that also influence musical practicing behavior.

### Summary and limitations

In summary, we demonstrated that musical aptitudes are positively correlated with each other as well as with intelligence, that these correlations are due to common genetic influences, and that, although part of the genetic covariation among the musical aptitudes was shared with IQ, a large part of the correlations among the aptitudes was due to genetic influences uniquely shared among the three musical aptitudes. The present sample was exclusively comprised of adult Swedish twins, and musical aptitude was operationalized as musical perceptual discrimination. Finally, it is possible that results would differ in younger or older populations or when using other measures of musical ability, such as instrument specific motor skills or musical achievement.
